# Identification of Vulnerable Populations and Areas at Higher Risk of COVID-19-Related Mortality during the Early Stage of the Epidemic in the United States

**DOI:** 10.3390/ijerph18084021

**Published:** 2021-04-12

**Authors:** Esteban Correa-Agudelo, Tesfaye B. Mersha, Adam J. Branscum, Neil J. MacKinnon, Diego F. Cuadros

**Affiliations:** 1 Department of Geography and Geographic Information Science, University of Cincinnati, Cincinnati, OH 45220, USA; diego.cuadros@uc.edu; 2 Health Geography and Disease Modeling Laboratory, University of Cincinnati, Cincinnati, OH 45220, USA; 3 Division of Asthma Research, Cincinnati Children’s Hospital Medical Center, Department of Pediatrics, University of Cincinnati, Cincinnati, OH 45220, USA; tesfaye.mersha@cchmc.org; 4 Department of Biostatistics, College of Public Health and Human Sciences, Oregon State University, Corvallis, OR 97331, USA; adam@oregonstate.edu; 5 Geospatial Health Advising Group, University of Cincinnati, Cincinnati, OH 45220, USA; nmackinnon@augusta.edu; 6 Medical College of Georgia, Augusta University, Augusta, GA 30912, USA

**Keywords:** COVID-19, ethnicity, neighborhood, health disparities, air pollution, comorbidity, healthcare capacity, multilevel models

## Abstract

We characterized vulnerable populations located in areas at higher risk of COVID-19-related mortality and low critical healthcare capacity during the early stage of the epidemic in the United States. We analyze data obtained from a Johns Hopkins University COVID-19 database to assess the county-level spatial variation of COVID-19-related mortality risk during the early stage of the epidemic in relation to health determinants and health infrastructure. Overall, we identified highly populated and polluted areas, regional air hub areas, race minorities (non-white population), and Hispanic or Latino population with an increased risk of COVID-19-related death during the first phase of the epidemic. The 10 highest COVID-19 mortality risk areas in highly populated counties had on average a lower proportion of white population (48.0%) and higher proportions of black population (18.7%) and other races (33.3%) compared to the national averages of 83.0%, 9.1%, and 7.9%, respectively. The Hispanic and Latino population proportion was higher in these 10 counties (29.3%, compared to the national average of 9.3%). Counties with major air hubs had a 31% increase in mortality risk compared to counties with no airport connectivity. Sixty-eight percent of the counties with high COVID-19-related mortality risk also had lower critical care capacity than the national average. The disparity in health and environmental risk factors might have exacerbated the COVID-19-related mortality risk in vulnerable groups during the early stage of the epidemic.

## 1. Introduction

Early Coronavirus Disease 2019 (COVID-19) data from Europe and Asia suggested high transmission and death rates. In late March of 2020, the United States (U.S.) had the fastest growing population impact across developed countries, leading to about 6,000,000 confirmed COVID-19 cases and 180,000 deaths by September 1. However, there was substantial spatial and temporal variation in the dynamics of the epidemic within the U.S., [1,2] with urban areas in New York, New Jersey, and Maryland experiencing the highest burden of infection early in the epidemic followed by a shift of the epicenter of the disease to rural areas in the South and Midwest late in 2020. Several countries including the U.S. have reported higher mortality rates (MR) for older people with comorbidities, including chronic lower respiratory diseases, diabetes, hypertension, and ischemic diseases among others [3,4,5,6].

Preliminary studies had started to create baseline population characteristics of COVID-19-related deaths during the early stage of the epidemic [7,8,9]. However, the roles of geospatial disparities, including sociodemographic and environmental exposures, and critical care capacity during the early stage of the epidemic are still not well understood. In general, identifying the population groups and areas at higher risk of infection and mortality during the early stage of an epidemic based on underlying health disparities and low critical healthcare capacity is a logical step to developing effective strategies for reducing the risk of infection and mortality throughout a country. Therefore, this study aims to: (a) assess sociodemographic and environmental drivers of COVID-19-related deaths and (b) identify vulnerable areas at higher risk of COVID-19 mortality but with low healthcare capacity during the early stage of the COVID-19 epidemic in the U.S. We use counts of confirmed deaths caused or contributed by COVID-19 to assess the impact of sociodemographic and environmental variables on the epidemic [10,11]. We hypothesized that during the early stage of the COVID-19 epidemic in the U.S., counties with predominantly minority populations, poor air quality, and low critical healthcare capacity were adversely affected by the epidemic.

## 2. Materials and Methods

### 2.1. Study Area and Data Sources

Counts of COVID-19-related deaths in the U.S. were obtained from a Johns Hopkins University database [12] for 3142 counties from all 50 states and the District of Columbia for the time period of 22 January to 1 September of 2020. This time period is the early stage of the epidemic in the U.S. that includes the initiation of intervention measures against the pandemic (e.g., reduced interactions and business closures) in March. Sociodemographic data were derived from the 2014–2018 American Community Survey (ACS) [13] and the Center for Disease Control (CDC) Social Vulnerability Index [14]. County-level comorbidities, including chronic lower respiratory disease (CLRD), diabetes mellitus, hypertensive diseases (HTA), and ischemic heart disease were obtained between 2010 and 2018 from U.S. Centers for Disease Control and Prevention [15]. Air pollution was quantified using surface annual particulate matter satellite images from 2000 to 2018 [16]. All data used in this study were recorded and analyzed at the county level.

### 2.2. Study Variables

The primary outcome variable in this study was COVID-19-related deaths. The projected expected number of deaths was calculated using an indirect standardization with the ACS standard population. We used the ratio between the number of COVID-19 deaths in a county and the number of deaths that would have been expected to occur based on a standardized population size. Demographic variables included the percent of the population in different age groups (<25, 25–34, 35–44, 45–59, 60–74, and ≥75 years), the percent of the population who self-identified as White, African American, or other races, and, following the Census Bureau definition of ethnicity [17], the percent of the Hispanic or Latino population. The percent of the population in poverty was obtained using the CDC’s Vulnerability Index [14]. We used 2000 to 2018 annual images of ground-level fine particle matter <2.5 μm (PM2.5) to assess the association between COVID-19-related mortality and long-term exposure of a county to several years of air pollutants [16]. These calibrated images are estimated at a 0.01° × 0.01° grid resolution combining satellite and monitoring stations data sources using a geographically weighted regression. We computed long-term exposure by averaging PM2.5 from 2010 to 2018 within each county (See Figure S1). We also calculated a regional air hub and road Connectivity Index for each county to examine the association between deaths and county-level airport hubs and main roads (See Figure S2). A categorical covariate with four geospatial connectivity levels was generated as follows: counties with an airport with more than 50,000 passengers per year (has an airport), counties next to a county with an airport (next to airport), counties crossed by a main road (crossed by a highway), and counties not neighboring a county with an airport and not crossed by a main road (no airport/highway). Due to the reported connection between COVID-19 mortality and underlying health problems [3,4,5,6,18,19], we included four chronic conditions as predictor variables for COVID-19 mortality. Crude county-level mortality rates per 100,000 people between 2010 and 2018 were included, obtained from the CDC Wonder database [15], including CLRD, diabetes mellitus, HTA, and ischemic heart disease. This study follows the guidelines of the Strengthening the Reporting of Observational Studies in Epidemiology (STROBE) [20]. Additional information about variables and STROBE checklist is included in the Supplementary Materials (See Table S1).

### 2.3. Multivariate Analyses of Risk Factors for COVID-19-Related Death

All covariates were selected according to an evidence synthesis process of preliminary reports [7,18,21,22,23,24]. A directed acyclic graph was built to infer causal effects to the observational data. We then removed open paths and checked for colliders and over control in the implied graph (See Figure S3) [25]. An exploratory data analysis of COVID-19 mortality rates was performed using linear regression for each covariate. For disease mapping, a Bayesian spatial Poisson model was used to estimate relative risks of COVID-19-related mortality [26,27]. Spatially correlated random effects were modeled as conditional autoregressive (CAR) priors using an adjacency county matrix (See Figure S4). We calculated a Moran’s I statistic to evaluate for spatial clustering of the residuals of the final model (See Figure S5). Quantile population classification was used to identify the 10 highest COVID-19 mortality risk areas in highly populated counties (4th quartile). A bivariate map combining COVID-19-related mortality risk and number of intensive care unit (ICU) beds was generated to identify areas with low critical healthcare capacity. ICU beds per 100,000 people were included as an index of critical healthcare capacity for each county [28]. All models were adjusted for age group due to the strong association between age and COVID-19 death. All numeric covariates were centered by subtracting the sample mean. The R language with the INLA, SpatialEpi, and raster packages was used for data analysis [29,30,31,32]. Detailed information is included in the Supplementary Materials 2 and 3.

## 3. Results

### 3.1. Sociodemographic and Socio-Environmental Variables

Table 1 presents descriptive statistics of COVID-19 deaths included in the study. The total number of deaths reported was 181,937, corresponding to 3.1% of the 5,958,655 COVID-19 confirmed cases. The highest standardized mortality ratios were found in NJ (SMR = 3.19; 15,950 deaths), NY (SMR = 2.98; 32,942 deaths), MA (SMR = 2.35; 9058 deaths), CT (SMR = 2.21; 4466 deaths), and LA (SMR = 1.83; 4821 deaths). Of the 3142 counties included in the study, 2460 had at least one confirmed COVID-19 death. The overall percent estimate of poverty was 15.6 (standard deviation (SD) 6.5) for the entire country. On average, the county-level percent of the population who were white was 83.0 (SD 16.7), while the average percent of African American was 9.1 (SD 14.6) and for other races, it was 7.9% (SD 10.2). For ethnicity, the average percent of Hispanic or Latino population was 9.3 (SD 13.9). The national average PM2.5 exposure was 8.0 µg/m (SD 2.4). For the Connectivity Index, 232 counties had an airport with more than 50,000 passengers per year, 1047 counties were next to airport hubs, 629 counties had a highway or main road, and 1200 counties are categorized as low transportation connectivity. The overall ICU beds capacity was 28.4 per 100,000 (SD 34.6). 

The COVID-19-related mortality rate per 100,000 people was higher in counties with high poverty (51.42 per 100,000), PM2.5 greater than 9.5 µg/m (50.75 per 100,000), and counties with an airport (42.23 per 100,000) (Figure S2). Counties with a high percentage of African Americans (60.43 per 100,000) and other races (54.95 per 100,000) had higher average mortality rates, whereas counties with a high proportion of white population had lower rates (15.82 per 100,000). Latino population also had a higher COVID-19-related mortality rate (40.55 per 100,000). Finally, counties with a high proportion of CLRD, HTA, and ischemic heart diseases had high COVID-19-related mortality rates of 51.42, 45.55, and 27.58 per 100,000 people, respectively. Detailed information is included in the Supplementary Materials 2 and 3 (See Figure S6 and Table S3)

### 3.2. Multivariate Analyses of Risk Factors for COVID-19-Related Death

Estimated relative risks (RR) of COVID-19 mortality from a Bayesian spatial Poisson regression analysis are presented in Table 2. Population over 75 years old or more was associated to 5% higher risk of mortality compared to population under 25 (RR = 1.05, 95% credible interval (CI): 1.01–1.08). For race, black and other races exhibit higher risk of COVID-19-related death compared to white population, 1% (RR = 1.01, CI: 1.01–1.02) and 2% (RR = 1.02, CI: 1.01–1.02) respectively. In ethnicity, the proportion of Hispanic or Latino population if infected with COVID-19 was associated with a 2% higher risk of COVID-19-related death (RR = 1.02, CI: 1.02–1.03). We found no association between the proportion of poverty and comorbidities and the risk of COVID-19-related death at county-level. For the long-term exposure to air pollution, we found that every additional unit of PM2.5 (1.0 µg/m) increased the risk of COVID-related death by 14% (RR = 1.14, CI: 1.08–1.20). Lastly, counties with an airport (RR = 1.31, CI: 1.14–1.51), and counties near to airports (RR = 1.13, CI: 1.03–1.24), had a higher risk of COVID-19-related death compared to counties with low transportation connectivity. More detailed information, including model formulation is included in the Supplementary Materials 3.

### 3.3. COVID-19 Disease Mapping

**Figure 1:**
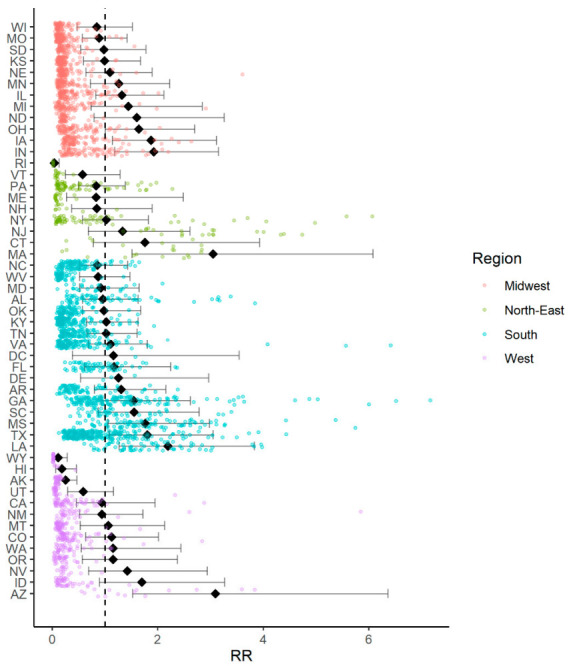
Relative risk for Coronavirus Disease 2019 (COVID-19) mortality rates by state.

Figure 1 and Table S4 illustrate the RR for COVID-19 at the state level. Seven of the 51 territories (50 states and D.C.) had a risk higher than average, namely, IA (RR = 1.87, 95% credible interval (CI): 1.13–3.11), IN (RR = 1.92, CI: 1.18–3.15), MA (RR = 3.05, CI: 1.51–6.08), LA (RR = 2.19, CI: 1.27–3.83), MS (RR = 1.76, CI: 1.05–2.97), TX (RR = 1.80, CI: 1.07–3.04), and AZ (RR = 3.08, CI: 1.52–6.34). Conversely, only four of the 50 states (RI, AK, HI, and WY) had an RR lower than the national average. 

**Figure 2:**
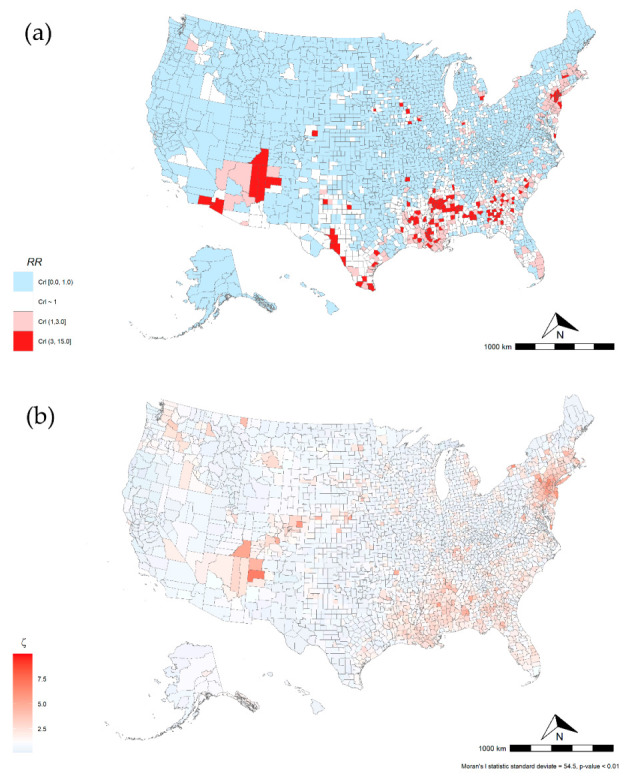
U.S. Relative Risk for COVID-19 mortality by county, mean = 0.63 (CrI range is 0.00–14.8) (**a**). U.S. COVID-19-related relative risk (RR) of death and (**b**) COVID-19 spatial effect.

Maps in Figure 2 illustrate the COVID-19 relative risk of death for all counties included in the study. Overall, we found that 315 counties from 33 states and D.C. had a higher risk of COVID-19-related mortality. Five states had 21 or more counties with high mortality risk, including GA (39), LA (42), MS (44), NJ (21), and TX (27). About 46% of the overall variance was explained by states, and nearly 44% was spatially structured indicating a spatial correlation of COVID-19-relative risk of death. This spatial pattern is present in Northeast and Southern states (Moran’s I *p*-value < 0.001). Table 3 shows the 10 highest COVID-19 mortality areas in highly populated counties (4th quartile). NY (4) and NJ (5) had nine of the top-10 highest risk locations. Six of 10 counties had higher poverty levels than the national county average (15.6%). These counties had on average a lower proportion of white population (48.0%) and higher proportions of black population (18.7%) and other races (33.3%) compared to the national averages of 83.0%, 9.1%, and 7.9%, respectively. Similarly, Hispanic and Latino population proportions were higher in these counties (29.3% vs. 9.3%). Nine of these 10 counties had a long-term PM2.5 exposure of at least 1.6 µg/m above the national average (8.0 µg/m). Nine of these 10 counties had an airport or were next to a county with an airport and 9 out of 10 had lower ICU beds availability than the national average of 28.4 ICU beds per 100,000 inhabitants. 

**Figure 3:**
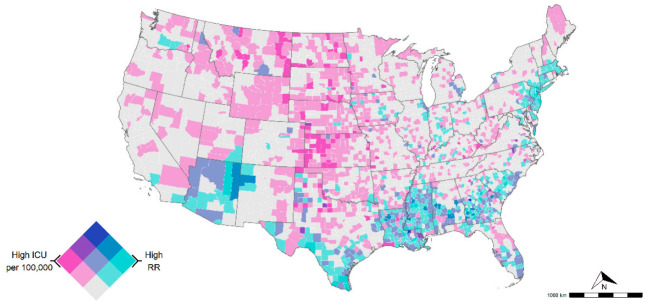
Intensive care units (ICU) bed availability per 100,000 (ICU information was not available in AK and HI). Dark purple indicates counties with high ICU availability and low mortality risk, whereas areas in darker green-blue indicate counties with high mortality risk but low ICU availability. Both variables were classified with a tertile scheme as follows: COVID-19-related RR (0–1 lower risk, 1–3 medium risk, and 3 > high risk) and ICU beds per 100,000 (<28.4 low availability, 28.4–100 medium availability, and >100 high availability).

Figure 3 illustrates a bivariate map of the COVID-19-related mortality risk and ICU beds availability for all counties in the conterminous U.S. There were counties with moderate to high mortality risk and low ICU availability in the Northeastern and southern regions. About 53% of these counties were in GA, LA, MS, NJ, and TX. In addition, areas with low risk of COVID-19-related mortality risk but high ICU availability were observed in KS, MT, ND, NE, and SD. Additional details can be found in the Supplementary Materials 4.

## 4. Discussion

This study provides state- and county-level characterization of the COVID-19-related mortality risk, including sociodemographic and socio-environmental factors during the early stage of the epidemic across the U.S. In addition, our study assessed the spatial link between COVID-19-related mortality risk and current critical healthcare capacity. Overall, we identified highly populated and polluted areas, regional air hub areas, race minorities (non-white population), and Hispanic or Latino population with an increased risk of COVID-19-related death during the first phase of the epidemic. The 10 most populated counties with the highest mortality risk had about fourfold higher risk than the national average with higher proportions of minorities residing in these counties. Finally, our spatial analysis highlights high-risk and low ICU availability areas that are worth noting for geographically targeted interventions, particularly during an early phase of an epidemic.

We found that the 10 highest COVID-19 mortality areas in highly populated counties (4th quartile) had about fourfold higher mortality risk than the national average during the early stage of the epidemic. Notably, these counties had on average, a lower white population and higher proportions of population in poverty, African American, other minorities, and Hispanic or Latino populations compared to their national averages. States and counties with a historically lower proportion of white population such as LA, NJ, and TX were at greater COVID-19-related mortality risk than other states. These demographic disparities in COVID-19-related mortality have been recognized in preliminary results of several major cities in other countries, including London [21,33,34]. Moreover, non-pharmaceutical interventions (e.g., school closing, physical distancing, lockdowns, and additional sanitation) are difficult to implement in these groups [35]. As a result, the effectiveness and benefit of these non-pharmaceutical interventions can be diluted by the work activities that involve person to person interaction and are more common in these low-income groups exposing them to a higher risk of infection and thus, higher mortality risk.

Air pollution was directly associated with higher COVID-19-related mortality risk. Of note, 8 of the top 10 counties with the highest mortality risk were very close to or above the cutoff for healthy air quality levels (PM_2.5_ of 12.0 µg/m), according to the U.S. national ambient air quality standards for particle pollution [36]. Air pollution is one of the leading risk factors for respiratory-related death globally [34], and this factor could be playing a key role in exacerbating the numbers of COVID-19-related deaths in highly polluted areas. Air pollution has an indirect impact on most of the organs and systems of the human body and indirectly comorbidities. Although we did not find significant associations between COVID-19 and comorbidities at the county level, air pollution has been identified as a contributing factor for many respiratory diseases like chronic obstructive pulmonary disease (COPD) [33,37], asthma [37,38,39,40,41,42], and lung cancer [43,44,45,46], which are concomitant comorbidities that had a strong association with COVID-19-related deaths at the individual level [3]. The health effects of air pollution depend on the components and sources of pollutants, which can vary among counties, seasons, and times. Initial evidence of incidence and mortality with comorbidities have been reported in Italy, with regional differences between the northern and southern region [8,23]. Although we found a strong positive association between air pollution and the risk of COVID-19-related death, the role of long-term exposure to poor air quality on COVID-19-related deaths in the U.S. is still not well understood, and thus more studies are needed, including for major U.S. cities taking into account long-term exposure to outdoor and indoor pollution. Furthermore, our results suggest that counties with airports have a higher COVID-19-related mortality risk than those with less connectivity. The lift of travel restrictions and high connectivity in these counties generated by airports can produce a high influx of imported infections that boost the local transmission of the virus in the county and consequently the number of COVID-19-related deaths.

According to our spatial analysis, 315 counties (10.0% of the total number of counties included in the study) from 34 states had a higher risk of COVID-19-related mortality than the national average. About 55% (173 out of 315) of these counties were located in five states: GA, LA, MS, NJ, and TX. Reasons for the increased mortality risk in these areas could be numerous. First, NJ shares borders with NY, which had experienced an intensive COVID-19 outbreak with about 5% of worldwide cases [47] and about 18% of U.S. deaths. Many people that work in the New York metropolitan area reside in NJ border counties, which might have contributed to the initial spread of the disease. Second, most of these states had a lower amount of white population and higher proportions of at least one minority group (African American and Latino) than the national average (See Table S4). State-average poverty was larger than the 15.6% study average for GA (20.7%), LA (22.0%), MS (24.1%), and TX (16.1%), but not NJ (10.2%). In terms of air pollution, four of these five states had long-term PM2.5 averages above the national average (8.0 µg/m), ranging from 9.4 µg/m (LA) to 10.6 µg/m (GA). For high populated and high COVID-19-related mortality risk counties, 9 of the 10 most populated counties were located in the New York metropolitan area, which is ranked 12 out of the 25 most ozone-polluted cities in the U.S. [48]. 

Our bivariate analysis showed that GA (22), LA (23), MS (21), NJ (18), and TX (17) represent 53% of the counties with moderate to high COVID-19-related mortality risk but lower critical care capacity than the national average of 28.4 ICU per 100,000 inhabitants. Most of the counties (79 out of 192) were also in areas with lower critical care capacity than the national average of 28.4 ICU per 100,000 inhabitants. With the onset of COVID-19 and the current lift of lockdown measures across the U.S., critical healthcare capacity might be potentially overwhelmed in several of these counties, not only with respect to ICU beds capacity but also in mechanical ventilators and staffing. Therefore, counties with high COVID-19-related mortality but low healthcare capacity identified in our study should be prioritized for strategies aimed at diminishing the overall number of COVID-19-related deaths, including patient relocation, strengthening of critical healthcare infrastructure and supply chains, and staff step-up [49].

Our study has several limitations worth noting. First, COVID-19 data that include comorbidity data are not available at the unit of analysis (county level). This issue might hamper precise comparisons of the real epidemic burden in specific groups and comorbidities. Second, we analyzed air pollution based on PM2.5 measures at county-level resolution. Smaller geographic areas and other pollutants including sulfate (SO4), nitrate (NO3), ammonium (NH4), organic matter (OM), black carbon (BC), mineral dust (DUST), and sea-salt (SS) would produce more refined inference [50]. Third, our Connectivity Index is based in a simplified geospatial attribute. Only airports and main roads were used as a Connectivity Index for each county. Other potential geospatial and population characteristics might influence the influx of travelers, and, therefore, the transmission dynamic. A further limitation relates to the challenges in translating cross-sectional associations into conclusions on causation of COVID-19-related deaths at county level. In addition, an ecological study is an approach for examining the association between factors and diseases by conducting analysis at the population level in specific areal units. In ecological studies, due to the lack of individual data, it is difficult to adjust for all potential confounding factors even if multivariate analysis is performed, leading to a possible ecological fallacy. Hence, our results should be interpreted with caution. Finally, this analysis only focuses on the early stage of this epidemic and results might not be comparable through time. Additional analysis focused on subsequent time periods and epidemic phases might reveal other disparities and dominant factors.

## 5. Conclusions

This study used geospatial approaches to examine population risk determinants of COVID-19-related deaths at the county level and identify vulnerable areas and populations at higher risk of COVID-19-related mortality during the early stage of the epidemic. The results have significant public health implications with respect to the critical healthcare infrastructure for an effective response to a pandemic, particularly during an epidemic’s early phase. The social gradient of health and environment in which underserved groups are highly vulnerable to more severe health outcomes can also be an important driver of the geographical and social disparity observed in the early stage of the COVID-19 epidemic in the U.S. Moreover, the healthcare capacity’s substantial regional disparities might increase the vulnerability of areas already at higher risk of disease spread during the early stage of the epidemic. Therefore, the results from this study can be used to guide the development of strategies for identifying locations and groups for targeted prevention efforts in vulnerable communities at high risk of disease spread and mortality during the early stage of an epidemic in the U.S.

## Figures and Tables

**Table 1:** Baseline characteristics.

COVID-19	n	
Confirmed Cases	5,958,655	
Confirmed Deaths	181,937	
**Sociodemographic**	**Mean**	**(SD)**
Age (years)		
Percent under 25	31.2	(4.8)
Percent 25–34	11.8	(2.3)
Percent 35–44	11.6	(1.5)
Percent 45–59	20.2	(2.2)
Percent 60–74	17.4	(3.7)
Percent 75+	7.8	(2.4)
Percent of population in poverty	15.6	(6.5)
Race		
Percent of white population	83.1	(16.9)
Percent of black population	9.1	(14.5)
Percent of other races	7.9	(10.2)
Ethnicity		
Percent of not Hispanic or Latino population	90.7	(13.8)
Percent of Hispanic or Latino population	9.3	(13.8)
**Crude mortality rates**	**Mean**	**(SD)**
Chronic lower respiratory disease	69.9	(26.0)
Diabetes mellitus	33.5	(14.7)
Hypertension	27.1	(16.9)
Ischemic heart disease	151.2	(57.2)
**Environment**	**Mean**	**(SD)**
Long-term PM2.5 exposure	8.0	(2.4)
Connectivity Index (n)		
Counties with no airport/highway	1200	
Counties crossed by a highway	629	
Counties next to airport	1047	
Counties with an airport	232	

**Table 2:** Estimated relative risks and 95% credible intervals from a Bayesian spatial Poisson regression analysis.

County-Level Covariates	RR	CrI: [2.5%, 97.5%]	
**Sociodemographic**			
Age			
Under 25	Ref	Ref	
25–34	0.98	(0.96	1.01)
35–44	1.01	(0.97	1.04)
45–59	1.02	(1.00	1.05)
60–74	0.98	(0.96	1.00)
75+	1.05	(1.01	1.08)
Percentage of population in poverty	1.01	(1.00	1.02)
Race			
Percent of white population	Ref	Ref	
Percent of black population	1.01	(1.01	1.02)
Percent of other races	1.02	(1.01	1.02)
Ethnicity			
Percent of non-Hispanic or Latino population	Ref	Ref	
Percent of Hispanic or Latino population	1.02	(1.02	1.03)
Crude mortality rates			
Chronic lower respiratory disease	1.00	(1.00	1.00)
Diabetes mellitus	1.00	(1.00	1.00)
Hypertension	1.00	(1.00	1.01)
Ischemic heart disease	1.00	(1.00	1.00)
Environment			
Long-term exposure to PM2.5	1.14	(1.08	1.20)
Connectivity Index			
Counties with no airport/highway	Ref	Ref	
Counties crossed by a highway	1.10	(1.00	1.20)
Counties next to airport	1.13	(1.03	1.24)
Counties with an airport	1.31	(1.14	1.51)

**Table 3:** Ten highest Coronavirus Disease 2019 (COVID-19) mortality risk areas in highly populated counties (4th quartile).

Location	Observed Counts	Expected Counts	Connectivity Index	PM25 (u/gml)	Poverty (%)	
Bronx, NY	4912	810	Next to airport	11.7	29.1	
McKinley, NM	243	41	Crossed by a highway	3.0	36	
Queens, NY	7224	1295	Airport	11.2	13	
Kings, NY	7290	1465	Next to airport	11.5	21.1	
Essex, NJ	2116	447	Airport	11.2	16.4	
Passaic, NJ	1245	284	Next to airport	9.6	16.7	
Union, NJ	1351	312	Next to airport	11.4	9.8	
Richmond, NY	1083	267	Next to airport	11.3	12.8	
Hudson, NJ	1508	377	Next to airport	12.3	16.3	
Bergen, NJ	2035	524	Next to airport	11.3	7	
**Location**	**ICU Per 100,000**	**White (%)**	**Black (%)**	**Other Races (%)**	**Latino (%)**	**RR CI: [2.5%, 97.5%]**
Bronx, NY	19.1	21.3	34.1	44.6	55.9	6.07 [5.90, 6.24]
McKinley, NM	35.7	15	0.7	84.3	14.3	5.85 [5.13, 6.61]
Queens, NY	6.4	39	18.3	42.7	28	5.58 [5.45, 5.71]
Kings, NY	10.8	43.5	32.6	23.9	19.2	4.98 [4.86, 5.09]
Essex, NJ	28.5	42.1	39.8	18.1	22.7	4.74 [4.54, 4.94]
Passaic, NJ	10.5	62.2	11.4	26.4	40.9	4.39 [4.15, 4.63]
Union, NJ	13.9	56.2	21.2	22.6	31.1	4.33 [4.11, 4.57]
Richmond, NY	15.2	74.3	10.2	15.5	18.3	4.06 [3.82, 4.3]
Hudson, NJ	13.3	55.1	12.4	32.5	43.2	4.0 [3.80, 4.21]
Bergen, NJ	13.1	71.4	6.0	22.6	19.4	3.88 [3.72, 4.05]

## Data Availability

Not applicable.

## Supplementary Materials

The following are available online at https://www.mdpi.com/article/10.3390/ijerph18084021/s1, Figure S1: U.S. 2000 to 2018 Long-Term Mean PM_2.5_ Concentrations by County, mean = 7.98 µg/m (range is 1.42–13.30), Figure S2: U.S. Connectivity index by county, Figure S3: Directed acyclic graph for Coronavirus Disease 2019 (COVID-19) mortality, Figure S4: U.S. counties adjacency matrix for the intrinsic CAR model, Figure S5: Bayesian spatial random effects (σ), Moran’s I statistic standard deviate = −4.61, *p*-value = 1, Figure S6: Exploratory data analysis covariates vs COVID-19 mortality rate. Table S1: Strobe checklist, Table S2: State Abbreviations List, Table S3: State summary for sociodemographic factors, Table S4: Relative risk by state.
