# Supplementary material for: Identification of Vulnerable Populations and Areas at Higher Risk of COVID-19-Related Mortality during the Early Stage of the Epidemic in the United States
Source: Int J Environ Res Public Health. 2021 Apr 12;18(8):4021. doi: 10.3390/ijerph18084021 (PMC8070560; doi:10.3390/ijerph18084021)
Supplement: Supplementary file 1 [file ijerph-18-04021-s001.pdf]

Supplementary Materials

Supplementary Material 1: STROBE checklist

TableS1. Strobe checklist.

STROBE item	Item No	Recommendation	Location in manuscript where items are reported
Title and abstract	1	(a) Indicate the study's design with a commonly used term in the title or the abstract	(a) Both in title and abstract (methods and findings section)
		(b) Provide in the abstract an informative and balanced summary of what was done and what was found	(b) This was done
		Introduction	
Background/rationale	2	Explain the scientific background and rationale for the investigation being reported	Introduction, paragraph 1, 2
Objectives	3	State specific objectives, including any prespecified hypotheses	Introduction, paragraph 2
		Methods	
Study design	4	Present key elements of study design early in the paper	Methods, paragraph 1-3
Setting	5	Describe the setting, locations, and relevant dates, including periods of recruitment, exposure, follow-up, and data collection	Methods, paragraph 1
Participants	6	(a) Give the eligibility criteria, and the sources and methods of selection of participants	Methods, paragraph 2
Variables	7	Clearly define all outcomes, exposures, predictors, potential confounders, and effect modifiers. Give diagnostic criteria, if applicable	Methods, paragraph 2-3

Data sources/ measurement	8*	For each variable of interest, give sources of data and details of methods of assessment (measurement). Describe comparability of assessment methods if there is more than one group	
Bias	9	Describe any efforts to address potential sources of bias	
Study size	10	Explain how the study size was arrived at	Appendix A
Quantitative variables	11	Explain how quantitative variables were handled in the analyses. If applicable, describe which groupings were chosen and why	Methods, paragraph 3. Appendix A
Statistical methods	12	(a) Describe all statistical methods, including those used to control for confounding	Methods, paragraph 4
		(b) Describe any methods used to examine subgroups and interactions	
		(c) Explain how missing data were addressed	
		(d) If applicable, describe analytical methods taking account of sampling strategy	
		(e) Describe any sensitivity analyses	
Results			
Participants	13*	(a) Report numbers of individuals at each stage of study—eg numbers potentially eligible, examined for eligibility, confirmed eligible, included in the study, completing follow-up, and analyzed	Results, paragraph 1

		(b) Give reasons for non-participation at each stage	
		(c) Consider use of a flow diagram	Appendix A
Descriptive data	14*	(a) Give characteristics of study participants (eg demographic, clinical, social) and information on exposures and potential confounders	Results, paragraph 1
		(b) Indicate number of participants with missing data for each variable of interest	NA
Outcome data	15*	Report numbers of outcome events or summary measures	Results, table 1
Main results	16	(a) Give unadjusted estimates and, if applicable, confounder-adjusted estimates and their precision (eg, 95% confidence interval). Make clear which confounders were adjusted for and why they were included	Results, paragraph 2. Appendix B
		(b) Report category boundaries when continuous variables were categorized	NA
		(c) If relevant, consider translating estimates of relative risk into absolute risk for a meaningful time period	NA
Other analyses	17	Report other analyses done—eg analyses of subgroups and interactions, and sensitivity analyses	Results, paragraph 3
		Discussion	
Key results	18	Summarize key results with reference to study objectives	Discussion, paragraph 1
Limitations	19	Discuss limitations of the study, taking into account sources of	Discussion, paragraph 5

		potential bias or imprecision. Discuss both direction and magnitude of any potential bias	
Interpretation	20	Give a cautious overall interpretation of results considering objectives, limitations, multiplicity of analyses, results from similar studies, and other relevant evidence	Discussion, paragraph 1-4
Generalizability	21	Discuss the generalizability (external validity) of the study results	Discussion, paragraph 6
Other information			
Funding	22	Give the source of funding and the role of the funders for the present study and, if applicable, for the original study on which the present article is based	Mentioned in the acknowledgements section.

Supplementary Material 2: Covariate selection criteria and definitions

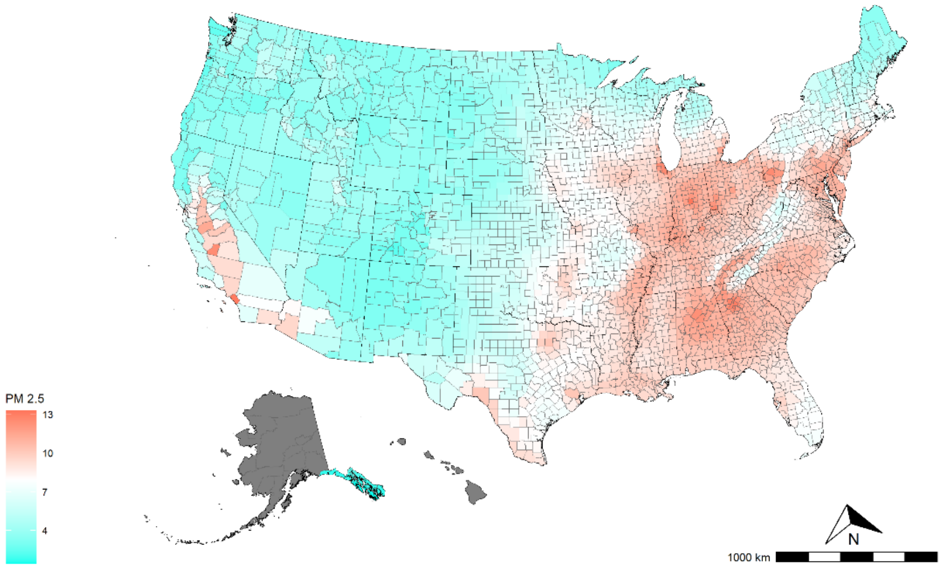

Figure S1. U.S. 2000 to 2018 Long-Term Mean PM2.5 Concentrations by County, mean=7.98 µg/m (range is 1.42-13.30).

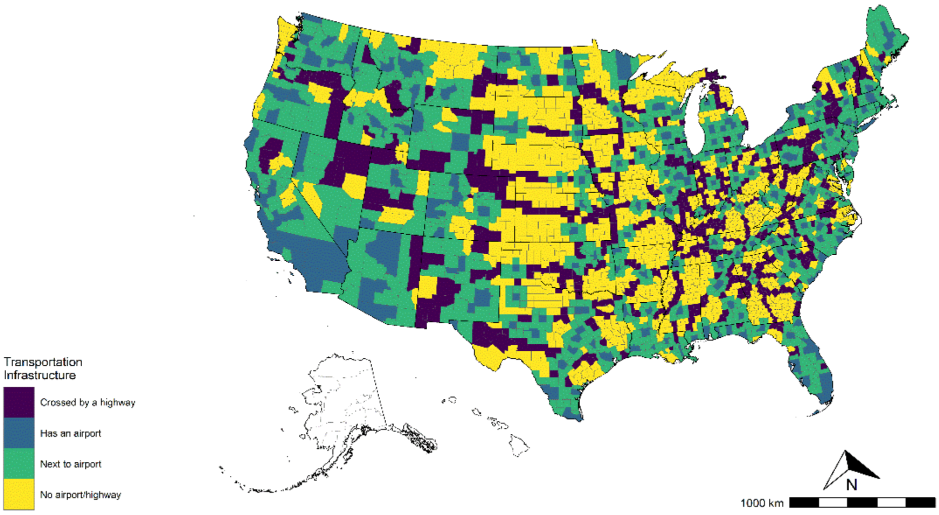

Figure S2. U.S. Connectivity index by county.

All covariates were selected according to an evidence synthesis process of relevant references [1–6].

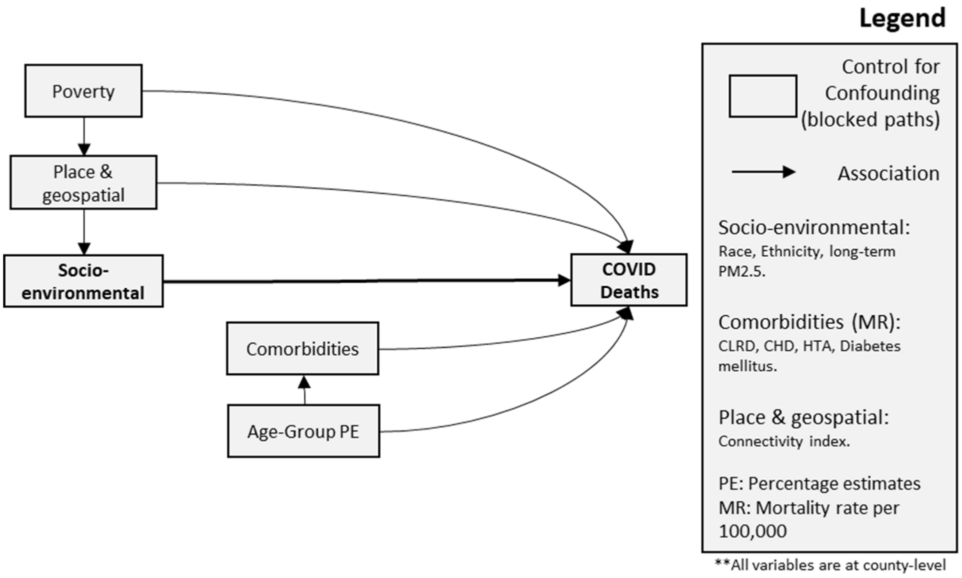

Figure S3. Directed acyclic graph for Coronavirus Disease 2019 (COVID-19) mortality.

The following variables were obtained from the 2014-2018 American Community Survey.

Age: Percent estimate of the population in the following age groups: under 25 years, 25 to 34 years, 35 to 44 years, 45 to 59 years, 60 to 74 years, over 75 years. Variable names: DP05_0005PE, DP05_0006PE, DP05_0007PE, DP05_0008PE, DP05_0009PE, DP05_0010PE, DP05_0012PE, DP05_0013PE, DP05_0014PE, DP05_0015PE, DP05_0016PE, DP05_0017PE.

Poverty: According to the U.S. Census Bureau, the income money threshold and the consumer Price Index (CPI-U). If a family's total income is less than the family's threshold, then every individual of that family is considered in poverty [7]. Variable name: S0601_C01_049E.

Race: Percent estimate of white, black. Variable names: DP05_0037PE, DP05_0038PE.

Ethnicity: Hispanic or Latino origin or not. Percent estimate of Hispanic or Latino population. Variable names: DP05_0071P.

Underlying cause of death: Four COVID-related underlying cause of death including Chronic lower respiratory diseases (ICD-10: J40-J47), diabetes mellitus (ICD-10: E10-E14), hypertensive diseases (ICD-10: I10-I15), and ischemic heart diseases (ICD-10: I20-I25) were extracted from the CDC Wonder database using the ICD-10 standard code [8].

PM2.5: For the exposure estimates, PM2.5 cross-validated exposure estimates were produced by van Donekelaar et al [9].

Table S2. State Abbreviations List.

State	Abbreviation
ALABAMA	AL
ALASKA	AK
ARIZONA	AZ
ARKANSAS	AR
CALIFORNIA	CA
COLORADO	CO
CONNECTICUT	CT
DELAWARE	DE
FLORIDA	FL
GEORGIA	GA
HAWAII	HI
IDAHO	ID
ILLINOIS	IL
INDIANA	IN
IOWA	IA
KANSAS	KS
KENTUCKY	KY
LOUISIANA	LA
MAINE	ME
MARYLAND	MD
MASSACHUSETTS	MA
MICHIGAN	MI
MINNESOTA	MN
MISSISSIPPI	MS
MISSOURI	MO
MONTANA	MT
NEBRASKA	NE
NEVADA	NV
NEW HAMPSHIRE	NH
NEW JERSEY	NJ
NEW MEXICO	NM
NEW YORK	NY
NORTH CAROLINA	NC
NORTH DAKOTA	ND
OHIO	OH
OKLAHOMA	OK
OREGON	OR
PENNSYLVANIA	PA
RHODE ISLAND	RI
SOUTH CAROLINA	SC
SOUTH DAKOTA	SD
TENNESSEE	TN

TEXAS	TX
UTAH	UT
VERMONT	VT
VIRGINIA	VA
WASHINGTON	WA
WEST VIRGINIA	WV
WISCONSIN	WI
WYOMING	WY

Supplementary Material 3: Bayesian spatial model

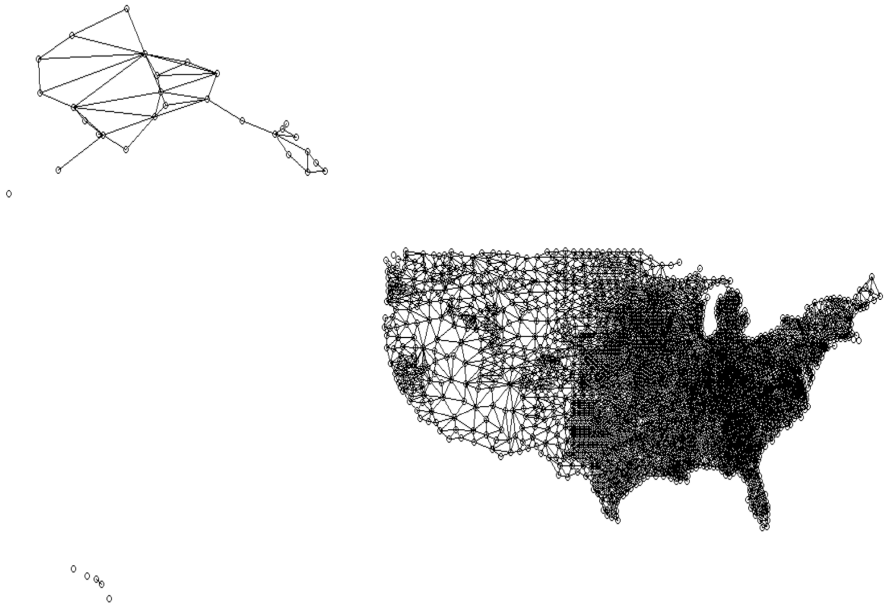

Figure S4. U.S. counties adjacency matrix for the intrinsic CAR model.

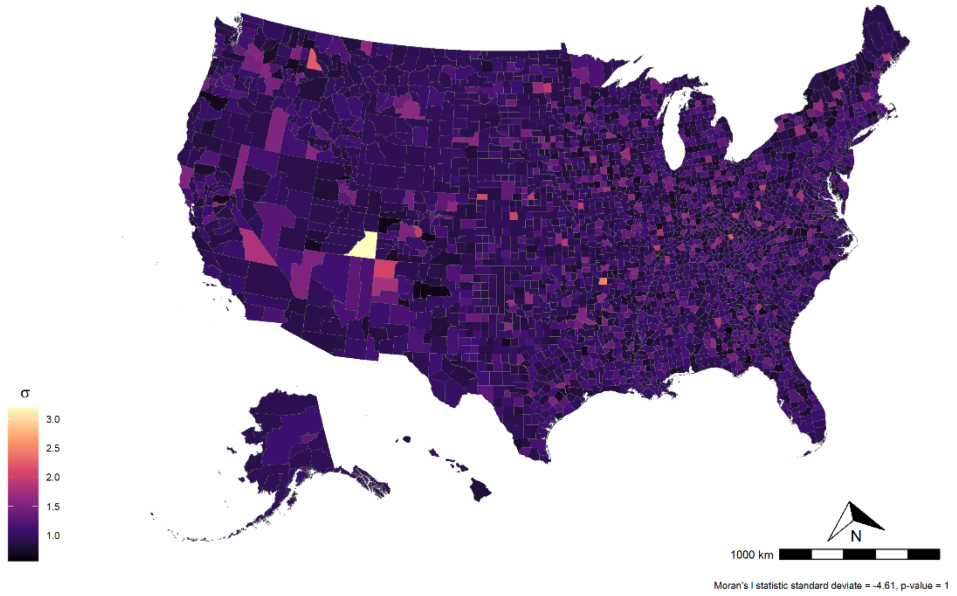

Figure S5. Bayesian spatial random effects (σ), Moran's I statistic standard deviate = -4.61, p-value = 1.We used the following Bayesian multilevel spatial regression model to estimate relative risks of COVID-related mortality at the county level.

$$Number\ of\ COVID19_{deaths} \sim Poisson(E * \theta), \quad (1)$$

where E denotes the expected number of deaths in the county, θ is the relative risk, and

$$log(\theta) = \beta_o + \beta_1 PM_{2.5} + \beta_3 Age_{25-34} + \beta_4 Age_{35-44} + \beta_5 Age_{35-44} + Age_{45-59} + \beta_7 Age_{60-74} + \beta_8 Age_{75+} + \beta_9 Black + \beta_9 OtherRaces + \beta_{10} Hispanic + \beta_{11} CLRD + \beta_{12} Diabetes + \beta_{13} HTA + \beta_{14} IHD + \beta_{15} Connectivity + \sigma_j, \quad (2)$$

Independent n (0,10) priors for each regression coefficient (β)

$$\sigma_j \sim halfCauchy(0,2), j = 1, \ldots, 50\ states + D.C, \quad (3)$$

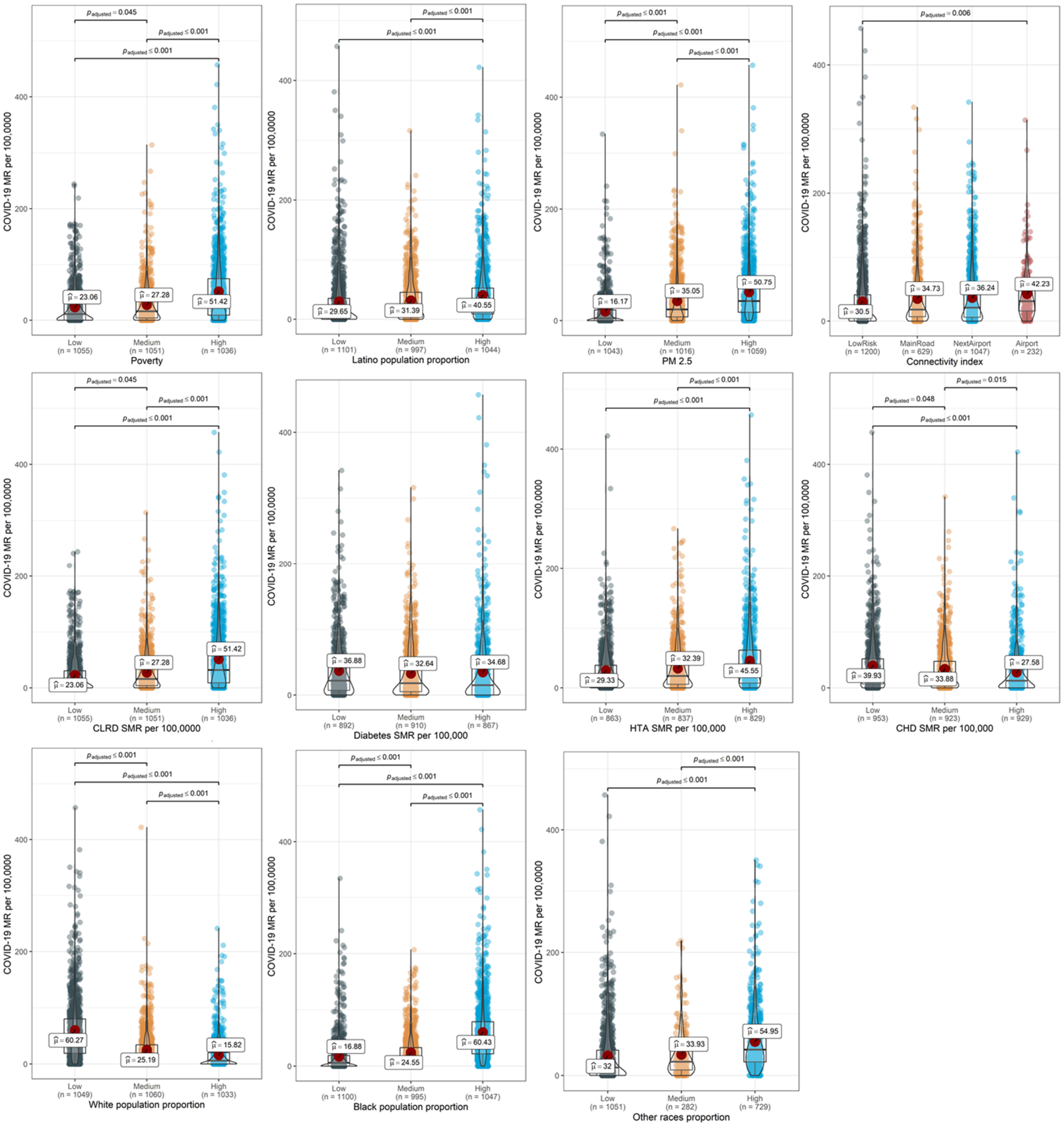

FigureS6. Exploratory data analysis covariates vs COVID-19 mortality rate.

Table S3. State summary for sociodemographic factors.

STATE	White (%)	Black (%)	Other Races (%)	Latino (%)	Poverty (%)	PM2.5 (u/gml)	ICU per 100,000
AK	50.8	1.3	47.9	5.2	13.3	1.8	NaN
AL	67.0	28.9	4.1	3.4	20.3	10.9	26.6
AR	78.2	16.2	5.6	5.2	19.8	9.0	22.4
AZ	74.6	2.0	23.4	31.1	20.0	5.0	23.6
CA	73.9	3.1	23.0	30.3	15.0	6.8	19.6
CO	90.7	1.6	7.7	20.1	13.1	4.0	28.1
CT	82.3	7.1	10.7	11.7	9.2	8.0	20.4
DC	41.0	46.9	12.1	10.9	16.8	12.0	59.5
DE	71.0	20.9	8.1	8.6	12.2	11.2	24.4
FL	79.1	14.5	6.4	14.0	16.6	8.9	25.3
GA	66.0	28.4	5.6	6.3	20.7	10.6	22.5
HI	28.8	1.4	69.8	9.5	10.8	NaN	NaN
IA	94.4	1.4	4.1	4.7	11.1	8.3	31.3
ID	91.8	0.3	7.9	12.8	15.0	4.5	22.8
IL	90.2	5.3	4.5	4.9	13.6	9.9	20.9
IN	93.0	2.8	4.2	4.0	12.9	11.0	20.4
KS	91.9	1.9	6.2	9.9	12.3	6.5	70.5
KY	93.2	3.6	3.2	2.4	21.0	10.1	23.1
LA	63.8	32.0	4.2	3.6	22.0	9.4	37.8
MA	83.2	6.2	10.6	9.1	10.7	7.4	24.1
MD	71.3	20.2	8.5	6.0	10.4	10.7	15.8
ME	95.2	0.9	3.9	1.5	13.8	4.7	21.2
MI	90.4	3.9	5.7	3.5	15.0	7.0	21.3
MN	91.3	1.8	6.9	4.4	10.8	6.6	33.8
MO	92.4	3.6	4.0	2.9	16.6	8.1	21.0
MS	55.4	41.6	3.0	2.4	24.1	9.5	29.1
MT	88.4	0.3	11.3	3.1	14.2	3.8	66.4
NC	72.3	20.4	7.3	7.2	17.5	9.6	24.5
ND	88.9	1.1	10.0	2.9	10.6	4.4	52.9
NE	94.4	0.9	4.7	6.7	11.0	5.5	43.2
NH	94.3	1.3	4.4	2.5	9.2	5.7	24.3
NJ	72.8	12.0	15.2	17.2	10.2	10.4	19.0
NM	78.2	1.4	20.4	47.7	21.1	3.9	21.7
NV	83.9	2.4	13.6	17.9	12.2	4.3	23.3
NY	85.0	6.3	8.7	7.8	13.6	7.6	19.3
OH	91.7	4.2	4.1	2.7	14.1	10.5	19.5
OK	75.3	3.5	21.2	9.1	17.0	7.6	36.5
OR	89.0	0.8	10.2	11.9	15.3	3.6	20.3
PA	90.6	4.8	4.7	4.3	12.6	9.4	27.2
RI	88.6	3.6	7.9	7.7	10.0	7.5	17.4
SC	59.6	35.7	4.7	4.5	19.4	10.4	20.0
SD	81.5	0.6	17.9	2.8	15.9	5.1	40.7
TN	88.8	7.4	3.9	3.5	17.9	10.0	20.1
TX	83.9	6.3	9.8	34.8	16.1	7.3	27.6
UT	90.8	0.5	8.6	9.1	11.9	4.3	21.5
VA	74.9	18.7	6.4	5.3	14.2	9.3	24.8

VT	95.3	1.0	3.7	1.8	11.3	5.6	17.1
WA	83.8	1.4	14.8	14.2	14.2	4.3	27.8
WI	91.4	1.7	6.9	3.7	11.5	7.3	21.4
WV	95.2	2.4	2.3	1.2	18.4	9.2	25.7
WY	92.6	0.5	6.9	8.2	11.5	3.8	39.1

Supplementary Material 4: Disease mapping

The model used for disease mapping of county-level data was:

$$Y \sim Po(E \times \theta) \quad (4)$$

$$\log(\theta_i) = \alpha + \sigma_i + u_i + v_i \quad (5)$$

where α denotes the overall risk level, σ is a state-level random effect, u is a spatially correlated random effect modeled as conditionally autoregressive, and v is a non-spatial random effect.

Table S4. Relative risk by state.

State	Region	RR, CI: [2.5%, 97.5%]			
**AK**	**West**	**0.25**	(0.13	,	0.46)
AL	South	0.96	(0.57	,	1.62)
AR	South	1.30	(0.80	,	2.15)
AZ	West	3.09	(1.52	,	6.36)
CA	West	0.94	(0.45	,	1.95)
CO	West	1.12	(0.63	,	2.01)
CT	North-East	1.75	(0.77	,	3.93)
DC	South	1.15	(0.38	,	3.54)
DE	South	1.25	(0.53	,	2.96)
FL	South	1.17	(0.61	,	2.24)
GA	South	1.54	(0.92	,	2.61)
HI	West	0.18	(0.07	,	0.45)
IA	Midwest	1.87	(1.13	,	3.11)
ID	West	1.70	(0.89	,	3.26)
IL	Midwest	1.31	(0.82	,	2.12)
IN	Midwest	1.92	(1.18	,	3.15)
KS	Midwest	0.99	(0.59	,	1.67)
KY	South	1.02	(0.65	,	1.62)
LA	South	2.19	(1.27	,	3.83)
MA	North-East	3.05	(1.51	,	6.08)
MD	South	0.92	(0.52	,	1.64)
ME	North-East	0.83	(0.27	,	2.48)
MI	Midwest	1.44	(0.73	,	2.84)
MN	Midwest	1.26	(0.72	,	2.22)
MO	Midwest	0.89	(0.56	,	1.41)
MS	South	1.76	(1.05	,	2.97)
MT	West	1.06	(0.53	,	2.13)
NC	South	0.85	(0.52	,	1.42)
ND	Midwest	1.60	(0.79	,	3.25)
NE	Midwest	1.09	(0.63	,	1.89)
NH	North-East	0.84	(0.36	,	1.89)
NJ	North-East	1.33	(0.68	,	2.61)
NM	West	0.94	(0.51	,	1.72)
NV	West	1.42	(0.69	,	2.94)

NY	North-East	1.02	(0.57	,	1.82)
OH	Midwest	1.64	(1.00	,	2.70)
OK	South	0.98	(0.57	,	1.68)
OR	West	1.15	(0.56	,	2.37)
PA	North-East	0.83	(0.50	,	1.38)
RI	North-East	0.04	(0.01	,	0.13)
SC	South	1.55	(0.87	,	2.78)
SD	Midwest	0.98	(0.54	,	1.77)
TN	South	1.02	(0.66	,	1.60)
TX	South	1.80	(1.07	,	3.04)
UT	West	0.58	(0.29	,	1.15)
VA	South	1.11	(0.68	,	1.80)
VT	North-East	0.57	(0.25	,	1.28)
WA	West	1.15	(0.54	,	2.43)
WI	Midwest	0.84	(0.47	,	1.51)
WV	South	0.87	(0.51	,	1.47)
WY	West	0.11	(0.03	,	0.28)

References

1. Niedzwiedz, C.L.; Donnell, C.A.; Jani, B.D.; Demou, E.; Ho, F.K.; Celis-Morales, C.; Nicholl, B.I.; Mair, F.; Welsh, P.; Sattar, N.; et al. Ethnic and socioeconomic differences in SARS-CoV-2 infection: Prospective cohort study using UK Biobank. *medRxiv* **2020**, *18*, 1-14, doi:10.1101/2020.04.22.20075663.
2. Williamson, E.; Walker, A.J.; Bhaskaran, K.J.; Bacon, S.; Bates, C.; Morton, C.E.; Curtis, H.J.; Mehrkar, A.; Evans, D.; Inglesby, P.; et al. OpenSAFELY: Factors associated with COVID-19-related hospital death in the linked electronic health records of 17 million adult NHS patients. *medRxiv* **2020**, doi:10.1101/2020.05.06.20092999. Available online: https://www.medrxiv.org/content/10.1101/2020.05.06.20092999v1 (accessed on 10 May 2020)
3. Halpin, D.M.G.; Faner, R.; Sibila, O.; Badia, J.R.; Agusti, A. Do chronic respiratory diseases or their treatment affect the risk of SARS-CoV-2 infection? *Lancet Respir. Med.* **2020**, *8*, 436–438, doi:10.1016/S2213-2600(20)30167-3.
4. Pansini, R.; Fornacca, D. Initial evidence of higher morbidity and mortality due to SARS-CoV-2 in regions with lower air quality. *medRxiv* **2020**, doi:10.1101/2020.04.04.20053595. Available online: https://www.medrxiv.org/content/medrxiv/early/2020/04/07/2020.04.04.20053595.full.pdf (accessed on 10 May 2020).
5. Fattorini, D.; Regoli, F. Role of the atmospheric pollution in the Covid-19 outbreak risk in Italy. *medRxiv* **2020**, *264*, 114732, doi:10.1101/2020.04.23.20076455.
6. Team CC-R. Preliminary Estimates of the Prevalence of Selected Underlying Health Conditions Among Patients with Coronavirus Disease 2019. MMWR Morb Mortal Wkly Rep; 2020;*69*, 382-386. doi:10.15585/mmwr.mm6913e2
7. Bureau C. How the Census Bureau Measures Poverty. U.S. Census Bureau, Available online: https://www.census.gov/topics/income-poverty/poverty/guidance/poverty-measures.html Accessed 05/11/2020 (accessed on 5 November 2020).
6. Centers for Disease Control and Prevention, National Center for Health Statistics. Underlying Cause of Death 1999–2018 on CDC WONDER Online Database, Released in 2020. Available online: http://wonder.cdc.gov/ucd-icd10.html (accessed on 26 April 2020).
7. van Donkelaar, A.; Martin, R.V.; Li, C.; Burnett, R.T. Regional Estimates of Chemical Composition of Fine Particulate Matter Using a Combined Geoscience-Statistical Method with Information from Satellites, Models, and Monitors. *Environ. Sci. Technol.* **2019**, *53*, 2595–2611, doi:10.1021/acs.est.8b06392.
